# The Assembly of Fish Gut Microbiomes Through Habitat Variation Provides Insight Into Redbelly Tilapia Invading a Large Subtropical River

**DOI:** 10.1002/ece3.70945

**Published:** 2025-02-11

**Authors:** Yaqiu Liu, Xinhui Li, Yuefei Li, Huifeng Li, Jie Li

**Affiliations:** ^1^ Pearl River Fisheries Research Institute Chinese Academy of Fishery Sciences Guangzhou China; ^2^ Guangzhou Scientific Observing and Experimental Station of National Fisheries Resources and Environment Guangzhou China

**Keywords:** assembly, gut microbiome, invasive tilapia, stochastic processes

## Abstract

The environment in which fish reside markedly shapes the composition of their gut microbiome. However, the precise mechanisms by which the assembly process of fish gut microbiota adapts to diverse habitat conditions remain largely uncharted, especially in the case of invasive tilapia, renowned for its remarkable adaptability to environmental changes. In this study, we employed high‐throughput 16S rRNA gene sequencing to explore the gut microbiome of redbelly tilapia from three distinct habitats. Our results showed substantial disparities in both the composition and diversity (alpha and beta) of the gut microbiome between wild and pond‐cultured redbelly tilapia. Notably, stochastic processes emerged as the dominant forces governing the assembly of the gut microbial community in redbelly tilapia. As the habitat shifted from pond‐cultured to wild, the influence of undominated processes in gut microbial community assembly waned, while the effect of dispersal limitation intensified. Co‐occurrence network analysis suggested that habitat variation contributed to the enhanced complexity of the gut microbial network in invasive tilapia during their transition from pond to wild environments. Across the three distinct habitats, variations were observed in the influence of environmental factors on the gut microbiota of tilapia. Moreover, our findings demonstrated that the gut microbiome of wild tilapia possessed unique characteristics, such as higher alpha diversity and a relatively greater abundance of genes encoding putative cellulolytic enzymes crucial for digesting the preferred food source of tilapia (hydrophytes), particularly in fragmented habitats with well‐developed cascade dams. Additionally, we identified that OTU8895 (*Clostridum_sensu_stricto_1*) and OTU11387 (*unclassified Bacilli*) were core biomarkers of the gut bacterial community in wild redbelly tilapia, offering valuable insights for the monitoring and management of invasive tilapia populations.

## Graphical abstract

In this study, we investigated the ecological processes that regulate the microbial community assembly of the redbelly tilapia gut among different habitats. Understanding invasive fish's gut microbial community assembly process can provide useful information concerning host plasticity mechanisms in altered aquatic habitats and offer new perspectives on the adaptation processes of invasive redbelly tilapia in large subtropical rivers.

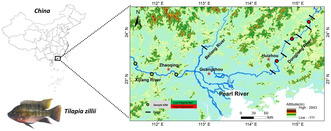

## 1 Introduction

Redbelly tilapia (*Coptodon zillii*) is introduced to China as a farmed species. Due to escapes and artificial releases, redbelly tilapia has widely invaded the Pearl River basin (Gu et al. 2020). Known for its high level of aggression, redbelly tilapia prefers slow‐flowing habitats and competes directly with native species for food resources and spawning sites (Geletu et al. 2024). Evidence shows that redbelly tilapia consumes a significant amount of hydrophytes, negatively impacting the ecosystem's structure. In subtropical rivers, tilapia also feed on plants essential for river and lake purification, which affects rehabilitation projects and leads to economic losses (Gu et al. 2018). Additionally, tilapia contribute to increased turbidity in aquatic habitats due to their feeding and excretion behaviors (Peterson, Slack, and Woodley 2005; Gu et al. 2015). Changes in turbidity within freshwater systems can alter nitrogen (N) and phosphorus (P) levels, subsequently affecting phytoplankton and periphyton biomass (Liu et al. 2008; Gu et al. 2018). The presence of tilapia in aquatic ecosystems can disrupt photosynthesis and overall productivity, which can result in eutrophication and modifications to the habitat (Liu et al. 2008; Martin, Valentine, and Valentine 2010; Russell, Thuesen, and Thomson 2012).

The Pearl River basin is primarily comprised of three major rivers: the Dongjiang River, the Xijiang River, and the Beijiang River. This area exhibits significant environmental heterogeneity, hosts vibrant fish populations, and boasts a rich diversity of species, including many endemics, ranking first among China's large rivers. Within the Pearl River basin, particularly in the Dongjiang River, numerous cascaded water conservancy projects have been established. The construction and operation of dams can disrupt downstream flow regimes, fluvial processes, river morphology, and ecological balance (Yang et al. 2024). Ongoing damming of these rivers fragments habitats and diminishes river connectivity (Almeida et al. 2019). As a result of dam construction, there has been a marked decline in rapid‐shoal habitats, with a notable decrease in natural river segments and a corresponding increase in lacustrine segments. This habitat fragmentation has significantly impacted the range and diversity of native fish species (Barbarossa et al. 2020). Moreover, the rising proportion of lacustrine segments within these river ecosystems has obstructed material and energy exchange while simultaneously creating favorable conditions for invasive species such as tilapia (Sun et al. 2023; Shuai, Li, and Lek 2023).

Fish that inhabit environments rich in bacteria have developed intricate symbiotic relationships. The composition of the gut microbiome is essential for the host's development and its capacity to adapt to significant environmental changes (Yan et al. 2016). From a macroecological perspective, the assembly of the gut microbiome is primarily influenced by deterministic and neutral (or stochastic) processes (Logue et al. 2011). The neutral theory posits that all individuals are ecologically equivalent, with stochastic processes predominantly shaping species dynamics and patterns through random birth/death rates, speciation/extinction events, and migration (Gravel et al. 2006). In recent years, a growing number of models have been created to elucidate the relative significance of deterministic and stochastic processes in the assembly of microbial communities (Ning et al. 2019). These models include the neutral theoretical model (NCM), normalized stochastic scale model (NST), Phylogenetic box‐based zero model analysis (iCAMP), Beta‐nearest taxon index (βNTI), and Raup Crick Index analysis (RCBray) (Mo et al. 2021; Ning et al. 2020; Zhou and Ning 2017). For instance, findings from microbial assembly mechanisms (βNTI and RCBray) indicate that non‐random processes are pivotal in regulating the gut microbial community assembly of wild large yellow crocea (Zhu et al. 2022). Therefore, gaining insight into the gut microbial community assembly process in invasive fish can offer valuable information regarding the mechanisms of invasion in natural aquatic habitats.

Thus, in recent years, heightened human activities, particularly related to water conservancy projects, have resulted in diminished river connectivity and the fragmentation of river habitats, a trend that has had a devastating impact on native fish species (Duan et al. 2009). Additionally, ongoing dam construction has created eutrophic and slow‐flowing environments that favor the proliferation of invasive redbelly tilapia (Shuai et al. 2019). Previous studies have shown that habitat changes significantly affect fish gut microbiota composition, alpha diversity, and beta diversity (Chen et al. 2021). However, the ecological processes that regulate the microbial community assembly of the redbelly tilapia gut among different habitats have yet to be comprehensively investigated. Thus, understanding invasive fish's gut microbial community assembly process can provide useful information concerning host plasticity mechanisms in altered aquatic habitats and offer new perspectives on the adaptation processes of invasive redbelly tilapia in the Pearl River.

## 2 Materials and Methods

### 2.1 Sample Collection

**FIGURE 1:**
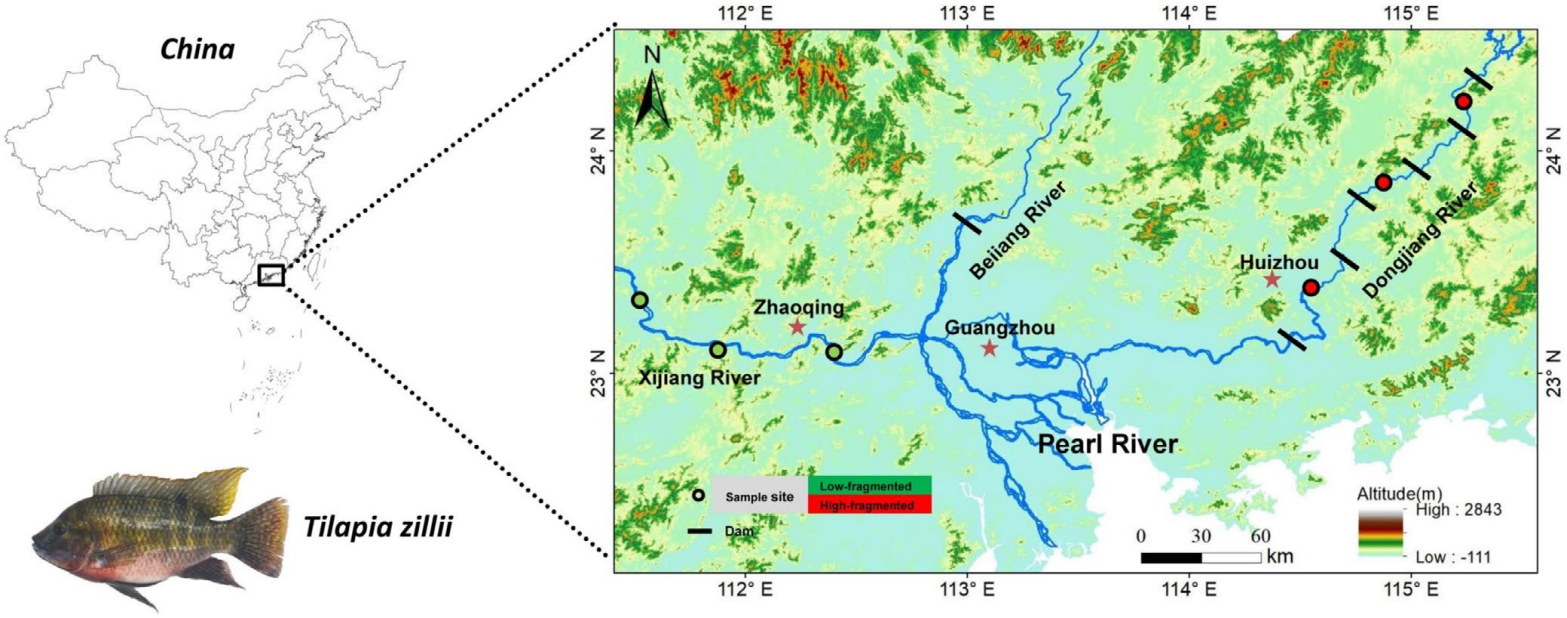
Sketch map showing the locations of sampling sites for *Coptodon zillii*.

A total of 75 fish gut samples were collected from three groups of redbelly tilapia in Guandong Province, China, including the Dongjiang River (DJ) group (*n* = 27), Xijiang River (XJ) group (*n* = 24), and Pond culture (PC) group (*n* = 24), during June to July 2022. The sample sites of wild fish are shown in Figure 1. Pond‐cultured samples were from the experimental culture ponds (23.069° N, 113.217° E). Water temperatures, dissolved oxygen (DO), and pH of sampling sites were measured with an HQ30 instrument (Hach Company, Loveland, CO, USA). Water samples were collected 0.5 m below the water's surface to determine nitrogen (TN) and phosphorus (TP). The TN and TP were determined by analyzing three duplicate samples collected at each water sample site (Pawlowski 1994). All the environmental parameters of the three groups are listed in Table S1. Fish body standard length (SL, to the nearest 1 mm) and body weight (Wt, to the nearest 1 g) were measured. DJ groups (SL: 150 ~ 182 mm; BW: 75 ~ 91 g); XJ groups (SL: 161 ~ 178 mm; BW: 79 ~ 93 g): PC groups (SL: 145 ~ 174 mm; BW: 80 ~ 87 g). A dose of MS 222 (3‐aminobenzoic acid ethyl ester methane sulfonate, Sigma, Germany) was administered to anesthetize all samples, and then they were quickly decapitated. All the instruments were sterilized prior to fish dissection. All gut content samples were immediately stored in liquid nitrogen. When returning to the laboratory, all the samples were transferred quickly to the −80°C ultra‐low refrigerator until further experiments.

### 2.2 DNA Extraction, Amplification, and High‐Throughput Sequencing

All samples were extracted at room temperature using a QIAamp DNA Stool Mini kit (Qiagen, Valencia, CA). The 16S rRNA genes from distinct regions were amplified using primers specific to the V3‐4 hypervariable region (341F–806R) (Caporaso et al. 2012). An extract of 1% agarose gel was used to purify the polymerase chain reaction (PCR) product and make it quantifiable utilizing Qubit 4.0 (Thermo Fisher Scientific, Waltham, CA) as directed by the manufacturer. The amplicons were pooled in equal quantities and sequenced using the Illumina PE250 platform (Illumina, San Diego, CA, USA) by standard protocols by Majorbio Bio‐Pharm Technology Co. Ltd., Shanghai, China, according to the standard protocols.

### 2.3 Bioinformatic Analysis

The 16S rRNA sequence data was processed employing the QIIME 1.9.1 software program developed by Caporaso et al. (2010). The paired‐end sequence reads were assembled using default settings and marginally filtered for quality. Operational taxonomic units (OTUs) were determined based on 16S rRNA gene sequences that had > 97% similarity. OTU classification was done by matching OTUs with the Silva132 database (Quast et al. 2012). Alpha diversity was assessed using Chao1, Ace, observed OTUs, and Faith's phylogenetic diversity (PD) indices. The Kruskal‐Wallis H test examined variations in alpha diversity indices of fish gut microbiomes across various habitats. Bar plots showed the average levels of phylum, family, and genus of gut bacteria (DJ, XJ, and PC). Linear discriminant analysis effect size (LEfSe) was employed to examine the variations in gut microbiota of redbelly tilapia from three different habitats (Segata et al. 2011). The gut microbial populations of *C. zillii* in three habitats were compared using permutational multivariate analysis of variance (PERMANOVA) with 999 permutations using the Bray‐Curtis distance metric. Nonmetric multidimensional scaling (NMDS) was used to visualize the results, according to Anderson (2010). The adonis function (PERMANOVA) in the vegan package was used in QIIME 1.9.1 to determine the proportion of variance attributed to species based on three different distances: Bray‐Curtis distance, unweighted UniFrac distance, and weighted UniFrac distance (Caporaso et al. 2010; Dixon 2003). A random forest‐supervised machine learning model was used to determine the significance of species differences between wild and farmed groups, as outlined by Ma et al. (2020). The model's accuracy was confirmed by receiver operating characteristic curve analysis by Giessen (2020). Additional indicator species analysis was used to identify biomarkers for distinct groupings. A distance‐based redundancy analysis examined the correlation between *C. zillii* gut bacteria and environmental factors. The research used the vegan package in R (Dixon 2003). We statistically assessed the impact of environmental factors on the gut microbial community by doing variance partitioning analysis using Monte Carlo permutation tests (999 permutations) in the vegan package (Dixon 2003). Phylogenetic Investigation of Communities by Reconstruction of Unobserved States (PICRUSt) was used to predict the microbial functions of redbelly tilapia gut samples across diverse environments. The 16SrRNA gene sequences were compared to the Kyoto Encyclopedia of Genes and Genomes (KEGG) database to create functional profiles of the microbial communities (Langille et al. 2013). A one‐way ANOVA was used to analyze KEGG pathways (level 2) of the redbelly tilapia gut microbiome among the three groups.

### 2.4 Microbial Community Structure Analysis

The significance of random processes in the formation of gut microbiome was assessed using a Sloan neutral model utilizing the R v3.5.1 code (Sloan et al. 2006; Chen et al. 2019). Gut microbial community stability was assessed by calculating the average variation degree (AVD) based on the departure from the average of the normally distributed OTU relative abundance across several environments (Xun et al. 2021). Subsequently, the null model analysis was conducted inside the bin. The study used the null model‐based phylogenetic metric beta nearest‐taxon index (βNTI) and the taxonomically modified Raup‐Crick metric (RC) of β‐diversity to assess phylogenetic and taxonomic diversity variance, as described by Zhou and Ning (2017). Each bin with a pairwise βNTI value larger than 2 or < −2 signifies heterogeneous or homogeneous selection, indicating a much higher or lower phylogenetic turnover than anticipated. The RC was then used to divide the remaining pairwise comparisons with absolute βNTI values ≤ 2 (Stegen et al. 2013; Zhou and Ning 2017). An RC value of more than 0.95 or < 0.95 indicates a significant departure from the null model predictions (Stegen et al. 2013). The proportion of pairwise comparisons where the absolute value of βNTI is ≤ 2 and RC is more than 0.95 was calculated as the impact of dispersion restriction. In contrast, those with the absolute value of βNTI ≤ 2 and RC < 0.95 were considered homogenizing dispersal. Only values of |βNTI| ≤ 2 and |RC| < 0.95 were considered to analyze the impact of drift or less dominating processes (Zhou and Ning 2017). As implemented in EcolUtils, Levins' niche breadth index was used to identify generalists and specialists of the microbiome communities (Salazar 2018). Generalists were defined as occurrences that exceeded 95% confidence intervals, while specialists were occurrences that fell below the 95% confidence intervals, and neutral species were those occurrences within the 95% confidence intervals (Wu et al. 2017).

### 2.5 Co‐Occurrence Network Analysis

Based on Spearman rank correlations with OTU abundance in redbelly tilapia gut bacteria, co‐occurrence networks were constructed. In the analysis of co‐occurrence networks, we employed statistically robust correlations (correlation coefficient *R* = 0.8; *p* < 0.001). It was determined to reduce the likelihood of false positive results in *R* 3.0 using the Benjamini and Hochberg FDR method (Benjamini, Krieger, and Yekutieli 2006). Then, Gephi 0.92 software was used to visualize networks and calculate network diagram parameters (e.g., node, edge) (Bastian, Heymann, and Jacomy 2009). In order to evaluate the relationship between the microbiome and environmental variables, Spearman rank correlations were used to construct co‐occurrence networks for the abundant phyla of the gut microbiome of the redbelly tilapia.

## 3 Results

### 3.1 Gut Microbiome Diversity and Composition of Redbelly Tilapia Altered With the Environment

**FIGURE 2:**
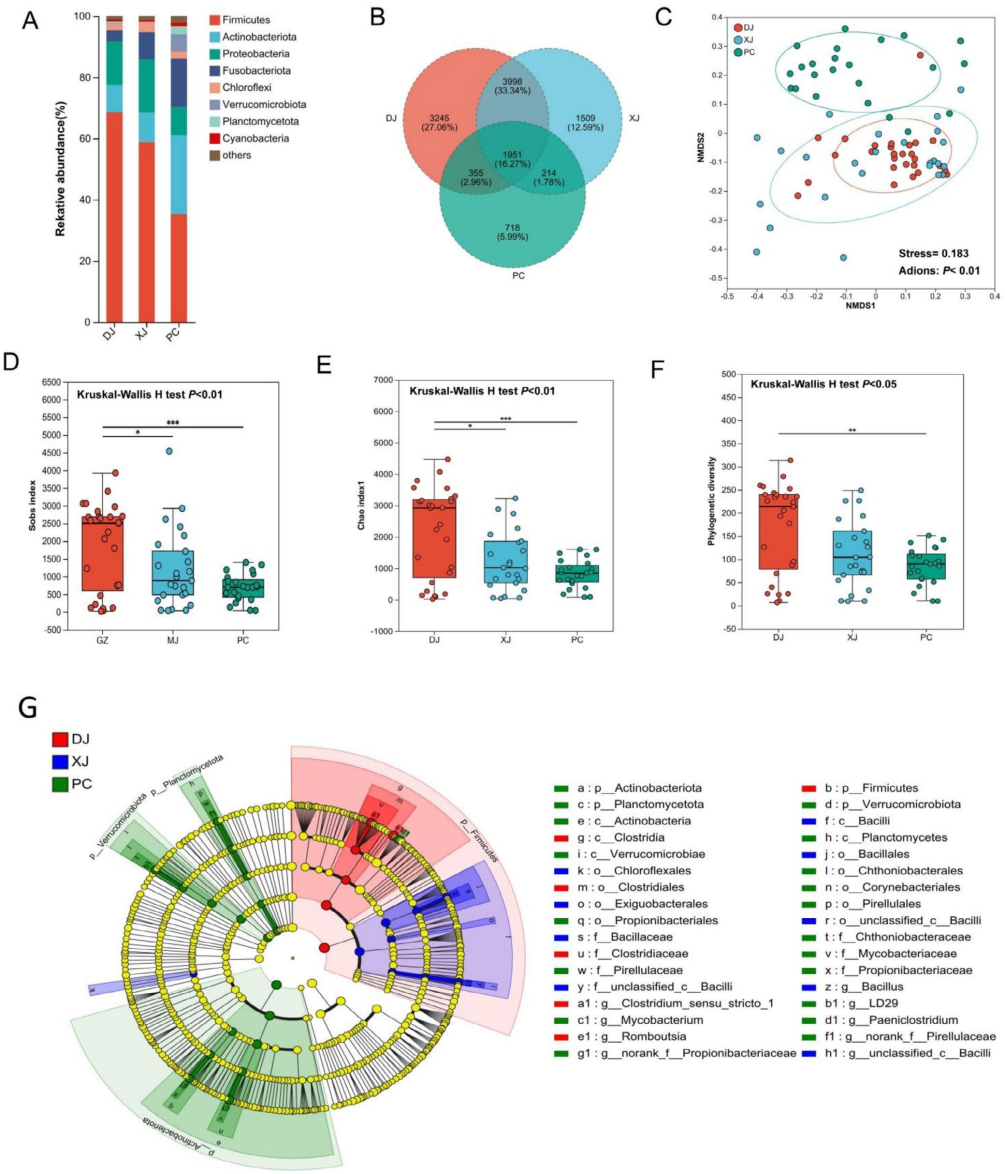
Differences in gut microbial composition and diversity among different habitats. (A) The phylum‐level relative abundance of the predominant microbes in gut samples collected from three distinct habitats. (B) The Venn diagram illustrated the quantity of unique and shared OTUs across three distinct habitats. (C) Analysis of nonmetric multidimensional scaling (NMDS) using Bray‐Curtis distances to compare gut samples from various habitats. (D, E, F) Alpha‐diversity index differences (Sob, Chao1, and phylogenetic diversity) that are statistically significant between samples: *p* < 0.05 (‘*’); *p* < 0.01 (‘**’); *p* < 0.001 (‘***’). (G) The results of linear discriminant analysis (LDA) effect size (LEfSe) indicated that the natural, fragmented, and pond habitats contained significantly more intestinal microbiota. LDA scores > 4 and *p* < 0.01 are displayed.

The dominant phylum in the DJ and XJ groups was Firmicutes (DJ: 71.05%; XJ: 58.23%), while Actinobacteriota (24.75%) was enriched in the PC group (Figure 2A). Apparent differences in the gut microbial composition (at the family and genus levels) of redbelly tilapia were also observed between wild and pond habitats (Figure S1). A Venn diagram was constructed to illustrate three groups' shared and unique OTUs in redbelly tilapia (Figure 2B). The DJ, XJ, and PC groups shared 1951 OTUs (16.27%). The PC group had the fewest unique OTUs (5.99%), whereas the group exhibited the largest number of unique OTUs (27.06%) (Figure 2B). NMDS analysis (Bray‐Curtis distance) identified a distinct separation between wild (DJ and XJ) and pond‐cultured samples, while no separation occurred between samples from DJ and XJ (Figure 2C). PERMANOVA (adonis) results indicated that the composition of the gut microbiome differed significantly among the three different habitats (*p* < 0.01; Figure 2C and Table 1). The Sob, Chao1, and PD indices of redbelly tilapia gut bacteria were significantly different between the DJ and PC groups (Kruskal‐Wallis H test, both *p* < 0.01) (Figure 1D–F). From XJ to DJ, we observed significant increases in the Chao1 and Sob indices (*p* < 0.05; Figure 1D,E).

**TABLE 1:** Permutational multivariate analysis of variance (PERMANOVA) results for the gut microbial community of tilapia zillii obtained from the various habitats.

Sample	PERMANOVA				
	Distance	df	*F*	*R* ^2^	*p*
Habitats	Bray‐curtis	2	9.2898	0.2051	0.001
	Unweighted‐unifrac	2	5.6329	0.1353	0.001
	Weighted‐unifrac	2	10.5072	0.2259	0.001

### 3.2 Specific Biomarkers of Gut Microbiota of Redbelly Tilapia Altered With the Environment

**FIGURE 3:**
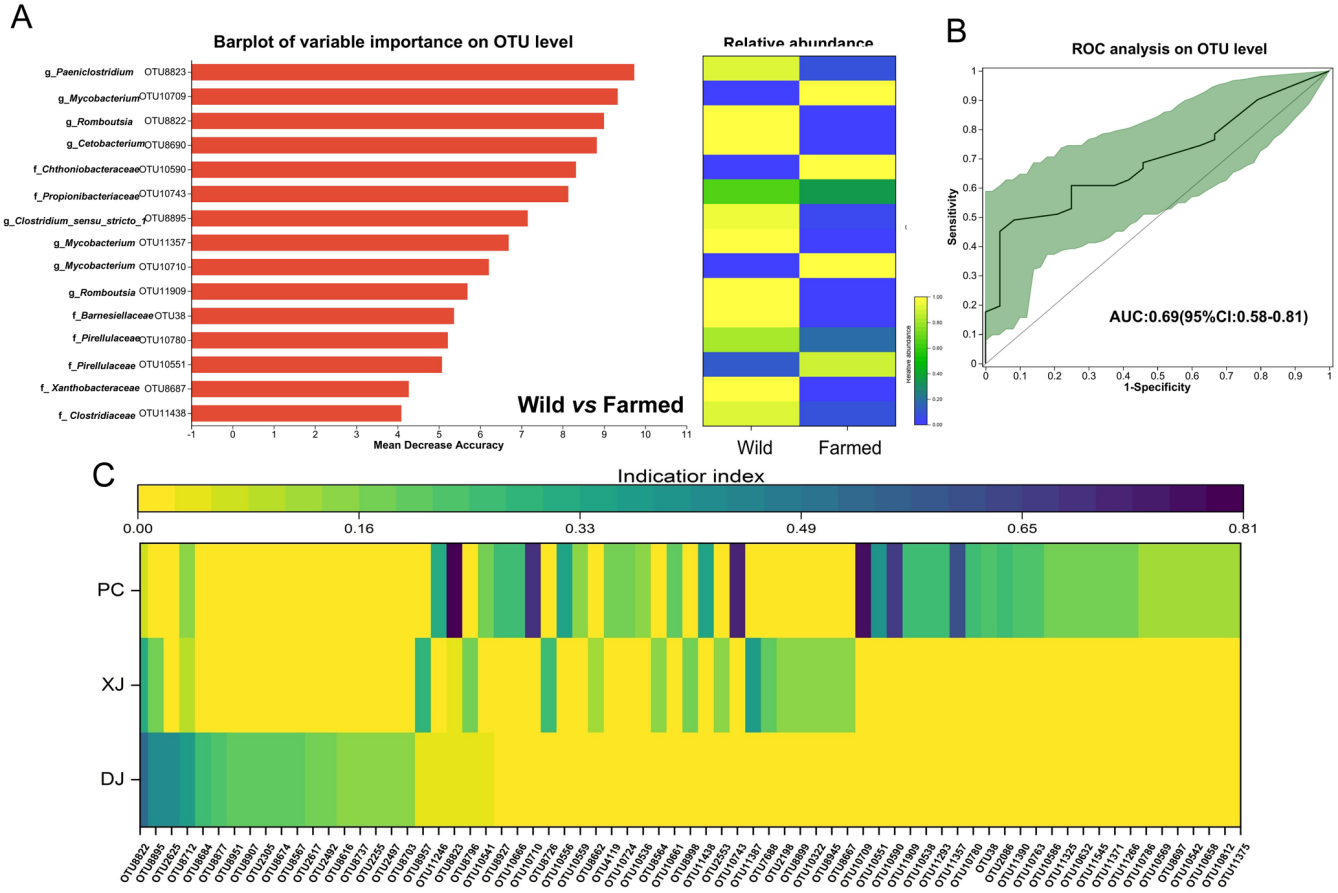
Origin predictions of gut microbiome of redbelly tilapia. (A) The importance and abundance distribution of key OTUs identified by the random forest model in differentiating redbelly tilapia habitats. (B) The efficacy of random forest models in classifying the gut microbiome of redbelly tilapia in pond and natural environments as measured by the AUC of ROC. (C) The indicator index was displayed in a heatmap across three different groups.

The differences in gut microbial communities were further characterized using LEfSe, which identified significant variations in the relative abundance of gut bacteria from distinct fish groups. The results showed that the phylum Firmicutes was significantly enriched in the DJ and XJ samples compared with the PC samples. In particular, we found that the genera *Clostridum_sensu_stricto_1* and *Bacillus* were significantly enriched in the DJ and XJ groups, respectively (Figure 2G). In contrast, the relative abundances of Actinobacteria, Planctomycetes, and Verrucomicrobiota were significantly higher in the PC samples than in the samples of the other two groups (Figure 2G). Moreover, an evaluation of the predictive ability of OTUs for interspecific differences between farmed (PC) and wild (DJ and XJ) tilapia was conducted using the random forest classifier. Our findings showed the top 15 most important OTUs, which were significantly important for discriminating wild and farmed groups (Figure 3A). Subsequently, classification accuracy was computed using the area under the curve (AUC = 0.69), verifying the accuracy of random forest prediction (Figure 3B). In addition, we identified biomarkers for three groups using indicator species analysis; our results showed significant differences in the indicative value of the same OTU for three groups of samples. Specifically, we found OTU8895 (*Clostridum_sensu_stricto_1*), OTU11387 (*unclassified Bacilli*), and OTU8823 (*Paeniclostridium*), which had the highest indicator indices in the DJ, XJ, and PC groups, respectively (Figure 3C).

### 3.3 Gut Microbial Community Assembly Processes Among Different Habitats

**FIGURE 4:**
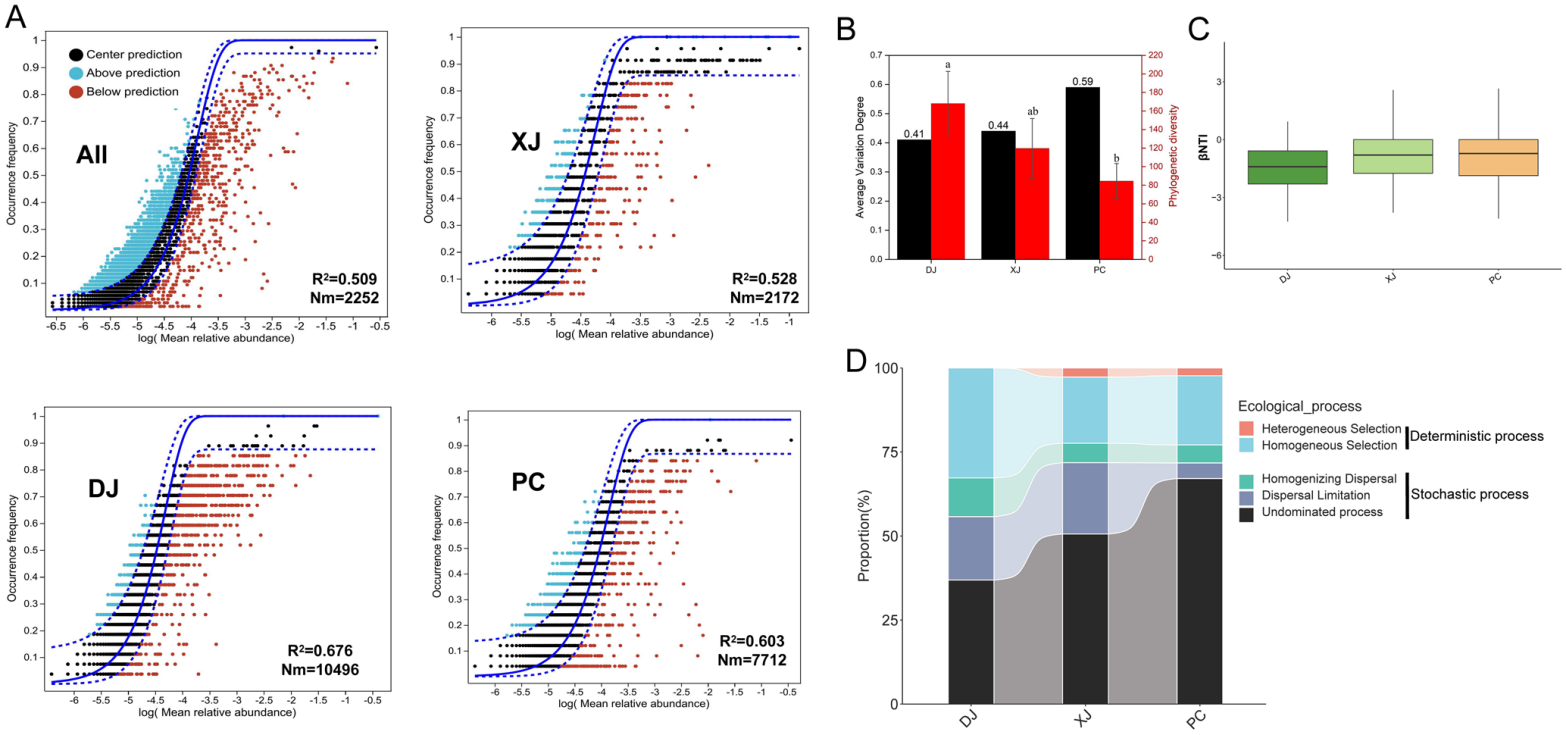
Assessing the randomness in the process of community formation in gut samples from natural, fragmented, and pond habitats. (A) Assessing the adequacy of the neutral community model (NCM) for community assembly. The solid blue lines depict the optimal fit of the neutral community model, while the dashed blue lines represent the 95% confidence interval around the model's prediction. OTUs falling within the predicted range are displayed in black, while those occurring more (blue) or less (red) than predicted by the NCM are shown in different colors. *R* ^2^ indicates the model's goodness of fit, whereas Nm is the result of multiplying community size by migration periods. (B) Phylogenetic diversity and average variation degree (AVD) of fish gut microbial communities in various settings. (C) A null model was used to examine the assembly process of primary microbial communities in gut samples. (D) The null model illustrates the impact of many ecological processes on the formation of the microbial community.

To evaluate the assembly processes of the gut microbiota in the tilapia of three different habitat types, the neutral community model was used for testing the significance of stochastic processes for all groups (Figure 4A). Our findings showed that stochastic processes dominated the community assembly of tilapia gut bacteria from three different habitat types (Figure 4A). Subsequently, further results indicated that the gut microbiota of the wild tilapia had low AVD and high PD values. Moreover, AVD values negatively correlated with PD at the OTU level (*r* ^2^ = 0.87, *p* < 0.01; Figure 4B).

The findings of βNTI also proved that stochastic processes were significant in determining the assembly of microbial communities of individuals from all groups (Figure 4C). Then, we quantified the contribution of different ecological processes to the assembly of fish gut microbial communities. In general, apparent differences in the contribution rate of homogeneous selection, dispersal limitation, and undominated processes of tilapia gut bacteria were found among the three groups (Figure 4D). Homogeneous selection (deterministic processes) had a higher contribution in affecting the assembly of the gut microbiota of tilapia from the three habitats than homogeneous selection (deterministic processes) (Figure 4D). The contribution rate of homogeneous selection for the DJ group gut microbiome was higher than that for the XJ and PC groups (Figure 4D). Notably, the contribution of dispersal limitation for the PC group gut microbiome was much lower than that for the XJ and DJ groups.

**FIGURE 5:**
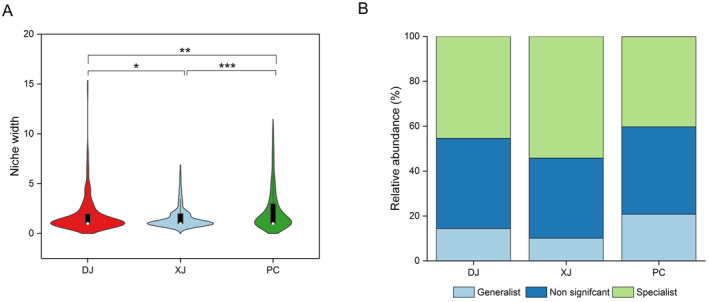
Relative abundance of niche width and the generalist and specialist ratio of the redbelly tilapia gut bacterial community. (A) Violin plots comparing the niche breadth of gut bacteria in redbelly tilapia across high‐fragmented (DJ), low‐fragmented (XJ), and pond (PC) habitats. Significant differences in niche width between different groups, *p* < 0.05 (‘*’); *p* < 0.01 (‘**’); *p* < 0.001 (‘***’). (B) Bar plots showing the relative contributions of generalists and specialists in the OTU levels of gut microbial communities among three different habitats.

Interestingly, the PC group exhibited the widest niche breadth among all the groups. In the wild, the DJ group had a greater niche breadth than the XJ group (*p* < 0.05; Figure 5A). In addition, the PC samples had the largest relative abundance of generalist bacteria, as seen in Figure 5B. The relative abundance of gut‐specialized bacteria decreased from XJ to DJ samples, whereas the relative abundance of generalist bacteria showed an opposite tendency (Figure 5B).

### 3.4 Ecological Networks of Gut Microbiota of Redbelly Tilapia Associated With Environmental Variables

**FIGURE 6:**
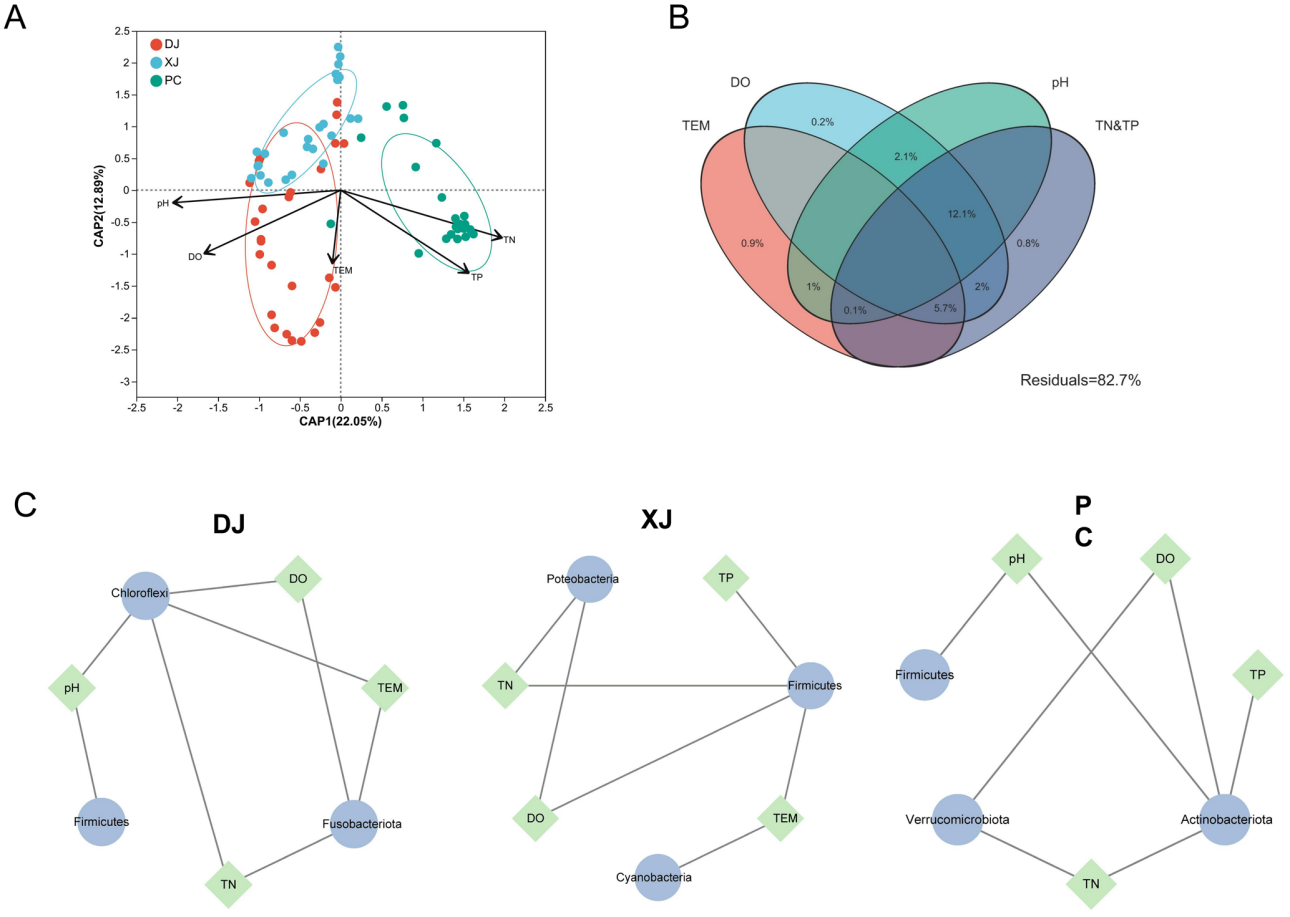
Relationship between environmental variables and redbelly tilapia's gut microbial community. (A) Distance‐based redundancy analysis (dp‐RDA) of gut bacteria of redbelly tilapia. The arrows show the direction and dimension of each microbial community at the OTU level. (B) Variance partitioning analysis (VPA) assesses the relative influence of TN & TP, pH, TEM, and DO groups on gut microbial community variance in three distinct environments. (C) Network analysis shows the connections between gut microbial populations and environmental factors. (D) The green circle symbolizes phylum, whereas the blue diamond symbolizes environmental variability. The lines suggest a correlation between bacteria and environmental factors. DO, dissolved oxygen; pH, water pH; TEM, water temperature; TN&TP, total nitrogen and total phosphorus.

Variations in the impact of environmental factors on tilapia gut microbiota were seen across the three disparate habitats. TN, DO, and pH were the primary environmental factors that affected the bacterial community composition of the DJ, XJ, and PC groups (Figure 6A,B). Then, we found the potential correlation between key bacteria species and environmental factors based on a two‐factor correlation network analysis. In the DJ network, Fusobacteria were influenced by TEM, DO, and TN, while Firmicutes were correlated with pH (Figure 6C). Chloroflexi were influenced by several environmental variables (i.e., DO, TN, and TEM) (Figure 6C). In the XJ network, several microbial phyla (Proteobacteria, Firmicutes, and Cyanobacteria) were influenced by environmental variables (i.e., DO, TN, TP, and TEM), while Cyanobacteria were influenced by TP (Figure 6C). In the PC network, three microbial phyla (Firmicutes, Actinobacteria, and Verrucomicrobia) were influenced by the environmental variables DO, pH, TN, and TP (Figure 6C).

Co‐occurrence networks were constructed by identifying bacteria species that exhibited significant correlations in the three groups (Spearman's coefficients *R* > 0.8 and *p* < 0.001; Figure S2). Then, we evaluated the network complexity of the gut microbial community across the three groups using node and edge parameters (Table S2). Overall, the gut bacteria of tilapia in the microbial network mainly collaborate instead of competing. Moreover, our findings showed a significant difference in the network between wild (DJ and XJ) tilapia and farmed (PC) tilapia. The microbial network of wild tilapia exhibited a greater weighted degree than the farmed tilapia, whereas the farmed tilapia exhibited a higher degree of network centrality betweenness (Table S2). Notably, the microbial network of DJ tilapia displayed a higher number of nodes than the XJ and PC tilapia.

### 3.5 Gut Microbiota Functional Differences Associated With Distinct Habitats

**FIGURE 7:**
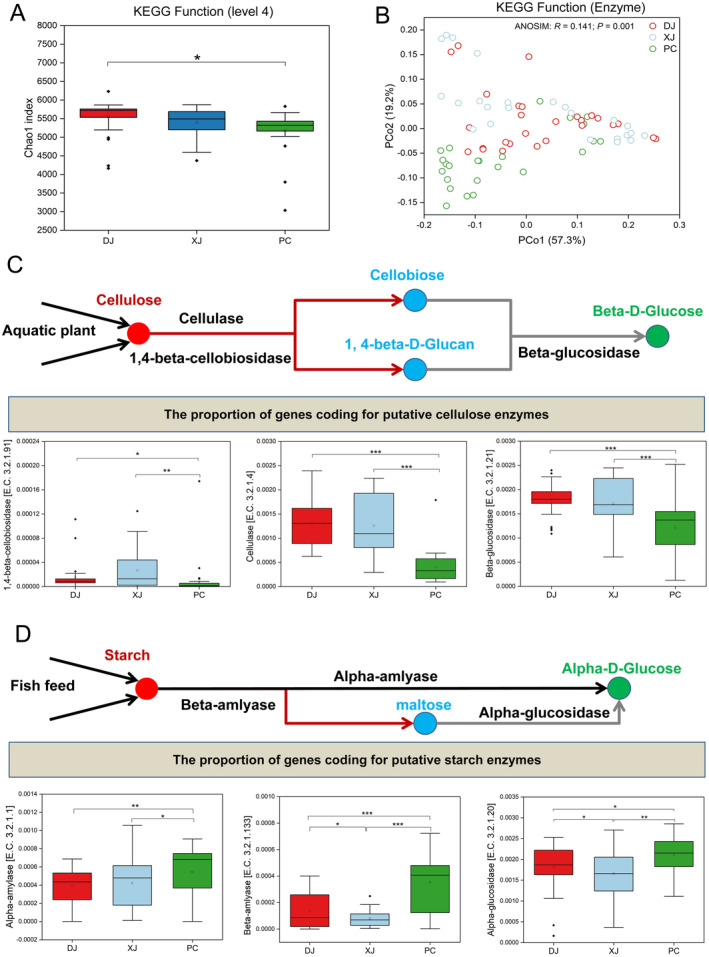
Function differences of gut microbiota community in the natural, fragmented, and pond habitats. (A) Boxplots displayed the divergence in alpha diversity (Chao1 index) according to KEGG function categorization. (B) Differences in beta‐diversity at the enzyme level in KEGG related to various habitats. Nonmetric multidimensional scaling (NMDS) using Bray–Curtis dissimilarity. (C) The boxplots display the proportions of coding genes for putative cellulose degradation enzymes in gut microbial communities and indicate the relevance of variations between groups. (D) Extent of putative starch degradation enzyme‐coding genes in gut microbial communities and the importance of group‐specific variations. Significant differences between groups were examined using the one‐way ANOVA test: *P* < 0.05 (‘*’); *p* < 0.01 (‘**’); *p* < 0.001 (‘***’).

Our results also indicated that wild and farmed redbelly tilapia gut bacteria differed significantly in terms of microbial function (level 2), e.g., amino acid metabolism, carbohydrate metabolism, lipid metabolism, and energy metabolism (Figure S3). The functional diversity of the gut microbiota in the three groups was evaluated using the Chao1 index based on KEGG functional gene annotation. The result indicated that the DJ group had a considerably higher Chao1 index than the PC group (*p* < 0.05; Figure 7A), suggesting a more diversified community function. Regarding KEGG‐level enzyme categories, significant differences in the functional pathways of the gut microbiota in the three groups were observed (ANOSIM *R* = 0.141, *p* = 0.001; Figure 7B). Tilapia preferred to feed on aquatic plants in the wild. Thus, we determined the relative abundance of three putative enzymes involved in cellulose use by gut microbes. There was a significant difference between groups for the average proportion of genes encoding putative 1,4‐beta‐cellobiosidase, cellulose, and beta‐glucosidase, and the abundance of genes in wild habitats (DJ and XJ) was significantly greater than that of pond‐reared samples (*p* < 0.01; Figure 7C). Further investigation indicated that the PC group had significantly higher relative abundances of alpha‐amylase and beta‐amylase than the wild (DJ and XJ) groups (*p* < 0.01; Figure 7D). In addition, there were significant differences in the mean proportions of genes coding for putative beta‐amylase and alpha‐glucosidase, where the DJ group had a higher proportion than the XJ group (*p* < 0.05; Figure 7C).

## 4 Discussion

The phenomenon of escape in redbelly tilapia farming is a significant factor commonly observed in natural river environments (Gu et al. 2020). The present study discovered notable differences in the gut microbial composition of redbelly tilapia between those cultivated in ponds and their wild counterparts. Previous research has established that variations in the composition of the fish gut microbiome can be found across different habitat types (Chen et al. 2021; Kim et al. 2021). Our findings indicated that the alpha diversity of the gut microbiome in wild redbelly tilapia inhabiting highly fragmented environments was significantly greater than that of their pond‐cultured counterparts. This aligns with our earlier research on Nile tilapia, which suggested that higher alpha diversity in the gut microbiome correlates with increased availability of diverse food resources in the habitat (Liu et al. 2023). Elevated gut microbiome diversity can enhance fish resilience to fluctuations in external resources and improve the efficiency of nutrient utilization from dietary sources (Escalas et al. 2021; Ghilardi et al. 2021; Liu, Li, Li, Li, and Zhu 2022; Liu, Li, Li, Zhou, and Li 2022). Furthermore, our findings indicated that redbelly tilapia transitioned from simpler pond cultures to more complex wild habitats, leading to significant alterations in their food composition. Changes in the dietary sources of tilapia are generally acknowledged as a key factor influencing the structure and function of gut microbial communities (Minich et al. 2022). In prior research, we showed that variations in habitat and fish feeding behavior have a direct impact on gut microbial communities (Liu, Li, Li, Li, and Zhu 2022; Liu, Li, Li, Zhou, and Li 2022). Furthermore, our results revealed significant differences between the gut bacteria of wild and farmed redbelly tilapia in terms of bacterial count (e.g., at the genus level) and microbial function (level 2), as shown in Figures S1 and S3. The observed differences between wild and farmed samples can likely be attributed to environmental changes and significant variations in food sources.

Fluctuations in environmental factors such as total nitrogen (TN), pH, and dissolved oxygen (DO) have significantly influenced the composition and diversity of the gut microbiome in tilapia. Generally, variations in habitat have led to changes in the gut bacterial community of tilapia. Research indicates that highly fragmented rivers have accumulated materials and energy, creating suitable slow‐flow habitats for tilapia (Shuai, Li, and Lek 2023). Our findings further demonstrated that redbelly tilapia exhibit strong adaptive capabilities in these highly fragmented (slow‐flow) environments, showing greater diversity in their gut microbiota compared to those in low‐fragmented or pond habitats. Moreover, we identified important biomarkers within the gut microbiota of redbelly tilapia across different habitats, which could serve as indicators for monitoring tilapia in varied environments. For example, *Clostridium_sensu_stricto_1* was significantly enriched in the gut microbiota of redbelly tilapia from fragmented habitats. In contrast, Bacillus was notably enriched in the gut microbiota of tilapia from low‐fragmented habitats. Additionally, in pond habitats, the relative abundance of Pirellulaceae in the gut microbiota of redbelly tilapia showed significant differences from wild samples. Previous studies have noted that redbelly tilapia primarily feed on large, abundant aquatic plants in their natural habitats (Gu et al. 2018). Both *Clostridium_sensu_stricto_1* and *Bacillus* present in aquatic animal guts are capable of degrading plant fibers and their secondary metabolites (Wang et al. 2021; Xiao et al. 2022).

Furthermore, we quantified the contribution of the assembly processes of the gut microbiota in the tilapia from different habitats. Our findings demonstrated that habitat diversity significantly influenced the community assembly processes of fish gut bacteria, consistent with previous studies (Yan et al. 2016; Zhu et al. 2022). Overall, the undominant process (stochastic processes) was the dominant factor regulating the redbelly tilapia gut microbial community assembly mechanisms among different habitats. In general, the neutral theory proposes stochastic processes, such as dispersal and undominant processes, which govern the assembly and shift of microbial communities (Ge et al. 2021). Dispersal limitation proved to be another critical factor affecting the gut microbial community in wild tilapia, while it was absent in pond‐cultured tilapia. This finding indicated that the reduced overlap of habitat space among wild tilapia leads to distinct gut bacterial communities (Lanan et al. 2016). Moreover, host immune systems and microbe‐filtering organs also modulate host neutrality‐based dispersal limitation. Generally, these findings imply that environmental selective pressures for tilapia may have caused differentiation of the gut bacterial communities among the different groups. The impact of dispersion limitation in gut bacteria increased, whereas the contribution rate of homogenous selection declined as the environment transitioned from low to high fragmented habitat. These findings may be due to tilapia's greater dispersal in a reservoir situated in an aquatic environment characterized by a comparatively low flow rate, as opposed to the low fragmented habitat (Shuai, Li, and Lek 2023). Previous research has demonstrated that habitat variation influenced and molded the assembly of aquatic microbial communities in a substantial way and mediated the equilibrium between determinism and stochasticity in the community assembly's ability to adapt to multiple environmental variables or selection pressures (Chen et al. 2019; Stadler and Del Giorgio 2022; Wang et al. 2020).

In addition, the niche width of tilapia gut bacteria increased from low to high fragmented habitats, which indicated that the dispersion of tilapia gut bacteria is more extensive in high fragmented rivers compared to low fragmented rivers. The variations in the biological activity of tilapia might account for this discovery. In suitable slow‐flowing environments, tilapia are more biologically and socially active and thus have a greater chance of sharing gut bacteria (Shuai et al. 2019; Gu et al. 2019). A previous study showed that the Dongjiang River, which is fragmented by many reservoirs, has experienced pronounced environmental disturbance (e.g., eutrophication) (Wei et al. 2023). The release of nitrogen‐phosphorus nutrients from sediments contributes to the eutrophication of reservoir water. For example, phosphates can contribute to the eutrophication of still or slow‐moving water (Zhou et al. 2023). Due to the high concentration of environmental bacteria in eutrophic water bodies, DJ individuals will consume more gut bacteria when consuming food. Additionally, the PC group had the greatest niche width of all three groups. Meanwhile, individuals in pond habitats tend to concentrate in a relatively small space, and gut microbiota are more prone to being transmitted between individuals. Overall, habitat variation significantly affects the niches of tilapia gut microbiota.

Relevant research has shown that environmental factors impact the gut microbiota of Nile tilapia, whose findings align with our results on redbelly tilapia (Bereded et al. 2022). The complexity of the redbelly tilapia gut microbial community network was impacted by habitat alteration. From pond to wild habitat, the complexity of the network played an increasing role in the microbial communities of the tilapia gut. The complexity of the network is crucial for comprehending the interrelationships between microorganisms (Shi et al. 2016). We found that the wild (DJ and XJ) groups exhibited higher numbers of network edges and degrees than the PC group, suggesting an increase in network stability in the wild environment. According to recent research, high network complexity suggests the necessity for a more stable microbial network to in withstanding interference from dangerous bacteria in the environment (Zhao, Guo, and Zhang 2021). As a result, the increase in microbial network complexity from pond to wild may assist tilapia withstand environmental stresses. We suggest that greater habitat heterogeneity in wild environments may increase microbiome diversity and stability, thereby enhancing the tilapia's ecological adaptability.

In this study, we observed the loss and gain of microbial taxa from wild to pond habitats. Gut microbiota in an enclosed pond habitat may experience a loss of gut bacteria and become populated by diverse species, maybe due to human interaction or the consumption of manufactured food. Differences in the composition of ingested foods are one of the most critical factors leading to changes in the gut microbiome. Our findings indicated a notable difference in KEGG function (enzyme level) diversity between wild and pond tilapia. Specifically speaking, there is a considerable rise in the presence of putative cellulose degradation enzyme‐coding genes in wild tilapia compared to pond tilapia. The results align with the study that redbelly tilapia prefer to eat substantial amounts of aquatic vegetation (Gu et al. 2018). Moreover, we found that redbelly tilapia maintained a high relative abundance of cellulose degradation enzyme‐coding genes in the DJ and XJ groups, suggesting that the feeding preference of redbelly tilapia for hydrophytes is relatively conservative in wild conditions. On the contrary, our research showed that the PC group exhibited a significantly elevated relative abundance of putative amylase‐coding genes. This finding implies that artificial rearing might simplify diets, which could impact the diversity and composition of gut microbiomes, particularly in herbivores that consume a high‐fiber diet in their wild habitat (Ley et al. 2008). Such a high‐fiber diet necessitates diverse complex microbial fermenters to metabolize digestion‐resistant feed (Ley et al. 2008). Studies have demonstrated that herbivores consume a limited variety of plants and consume low amounts of fiber, resulting in a low intake of short‐chain fatty acids (SCFAs) and low energy expenditure (Bornbusch et al. 2022). In pond conditions, calorie‐dense foods such as starch may not be able to compensate for the decrease in SCFAs from the loss of dietary fiber, resulting in adverse health effects (Warne and Dallas 2022). This study found that *Clostridium_sensu_stricto_1*, closely related to host SCFA production, was significantly enriched in the wild group (particularly in the DJ group). In addition to improving the health of redbelly tilapia, a variety in the diet results in an increase in SCFA‐producing microbial groups. According to these findings, certain gut microbiomes (high alpha diversity and possible benefits in food utilization) could be advantageous for redbelly tilapia in the wild, particularly in highly fragmented rivers, to survive, adapt, and invade.

## 5 Conclusion

The present study provides new insights into the adaptation processes of invasive redbelly tilapia. We investigated the gut microbiomes of these fish, comparing their composition across various aquatic habitats. Wild tilapia inhabiting fragmented environments exhibited high alpha diversity and a significant proportion of genes associated with enzymes involved in plant fiber digestion. This suggests that the redbelly tilapia's preference for hydrophytes remains consistent in natural settings. Additionally, our findings indicated that stochastic processes played the most significant role in assembling the gut microbial community of redbelly tilapia. Habitat variation notably influenced the mechanisms of gut microbial community assembly in these fish. We observed lower average values of diversity and higher phylogenetic diversity within the gut microbial communities of redbelly tilapia in wild habitats compared to those in ponds. This implied that the gut microbiomes of wild tilapia, particularly in fragmented river systems, tend to be more stable and resilient to perturbation. The results indicate that fragmented rivers, which contain slow‐flowing nutrients, significantly shape the gut microbial communities of redbelly tilapia. The high diversity of these communities may enhance local adaptation and facilitate the invasion of redbelly tilapia. Future research should focus on the impact of gut microbiota on the population expansion and adaptation of wild tilapia in subtropical river systems.

## Author Contributions

**Yaqiu Liu:** data curation (lead), formal analysis (lead), investigation (lead), methodology (equal), validation (equal), visualization (equal), writing – original draft (lead). **Xinhui Li:** supervision (equal), validation (equal), writing – review and editing (equal). **Yuefei Li:** investigation (equal). **Huifeng Li:** resources (equal), software (equal). **Jie Li:** funding acquisition (equal), supervision (equal).

## Ethics Statement

The methods involving animals in this study were conducted in accordance with the Laboratory Animal Management Principles of China. All experimental protocols were approved by the Ethics Committee of the Pearl River Fisheries Research Institute, Chinese Academy of Fishery Sciences.

## Conflicts of Interest

The authors declare no conflicts of interest.

## Supporting information


Data S1.

## Data Availability

Sequencing data and relevant files have been uploaded to the Sequence Read Archive with the accession number PRJNA1105451.
