# Supplementary material for: The Assembly of Fish Gut Microbiomes Through Habitat Variation Provides Insight Into Redbelly Tilapia Invading a Large Subtropical River
Source: Ecol Evol. 2025 Feb 11;15(2):e70945. doi: 10.1002/ece3.70945 (PMC11814482; doi:10.1002/ece3.70945)
Supplement: Supplementary file 1 — Data S1. [file ECE3-15-e70945-s001.doc]

**SUPPLEMENTARY MATERIALS**

**A**

**
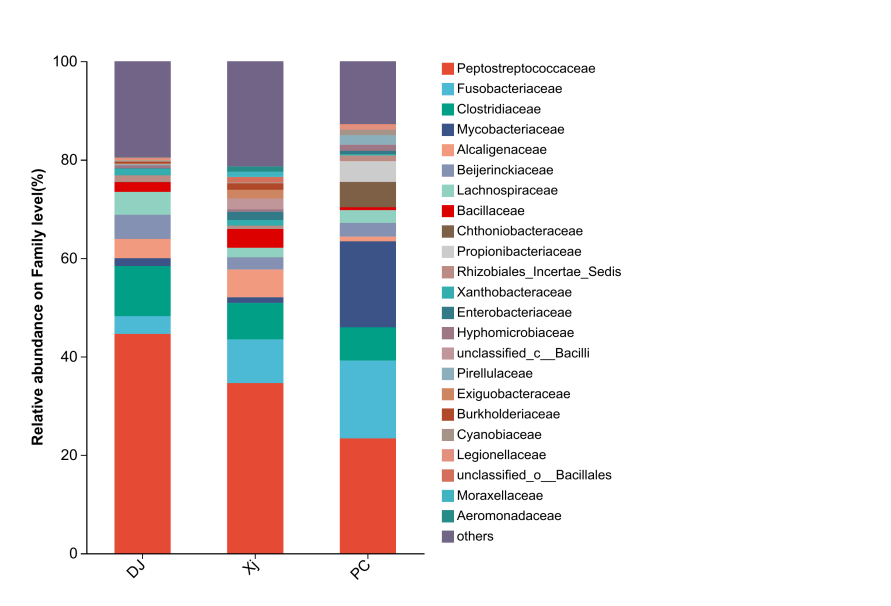
**

**B**

**
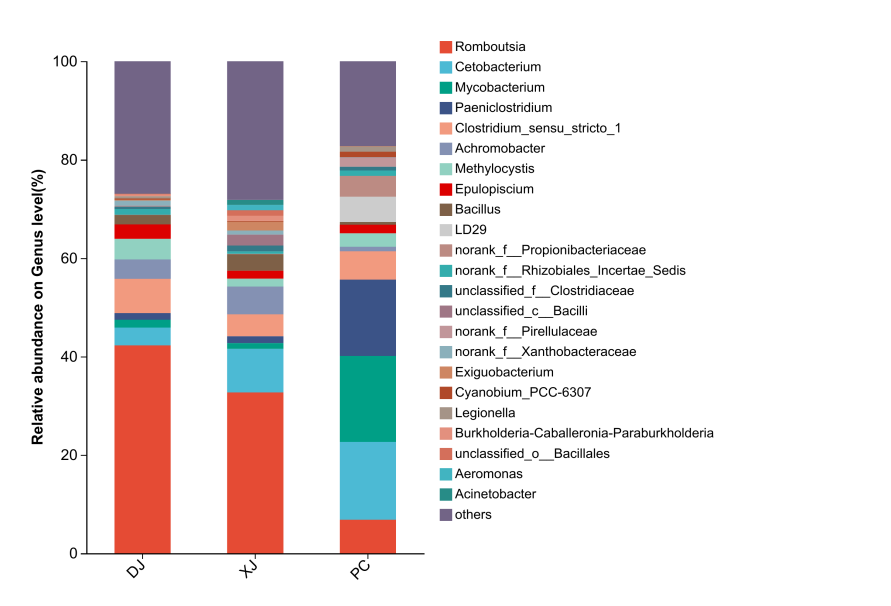
**

Figure S1 Comparison of the microbial community of redbelly tilapia in the different relative gut length groups. Dominant gut microbiota composition in the different groups at the family (A) and genus (B) level; each bar represents average relative abundance of each microbial taxon within a group at the family and genus level.

**
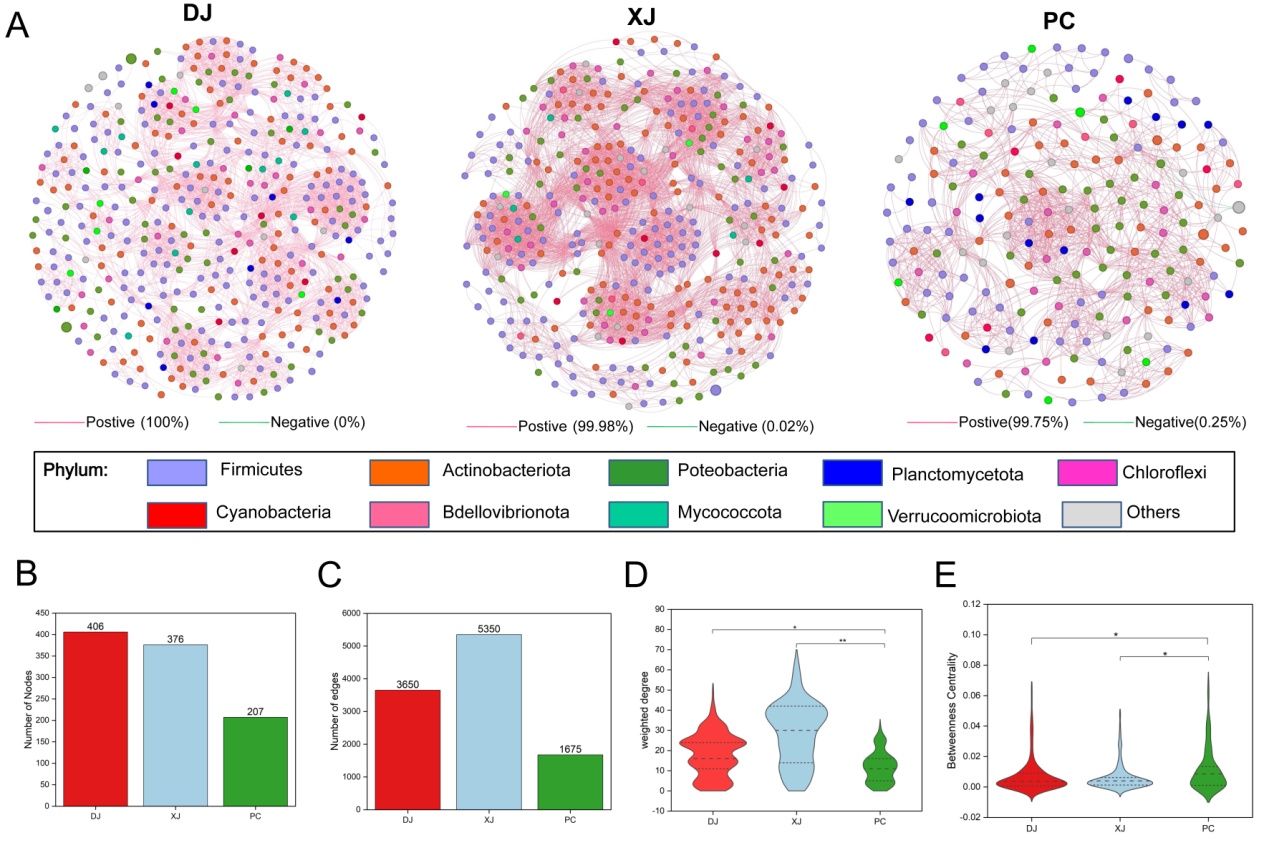
**

Figure S2 Co-occurrence networks of gut micrbiome of redbelly tilapia in the high fragmented (DJ), low fragmented (XJ), and pond (PC) habitats. Nodes symbolized the operational taxonomic units (OTU), whereas edges denoted substantial association.

**
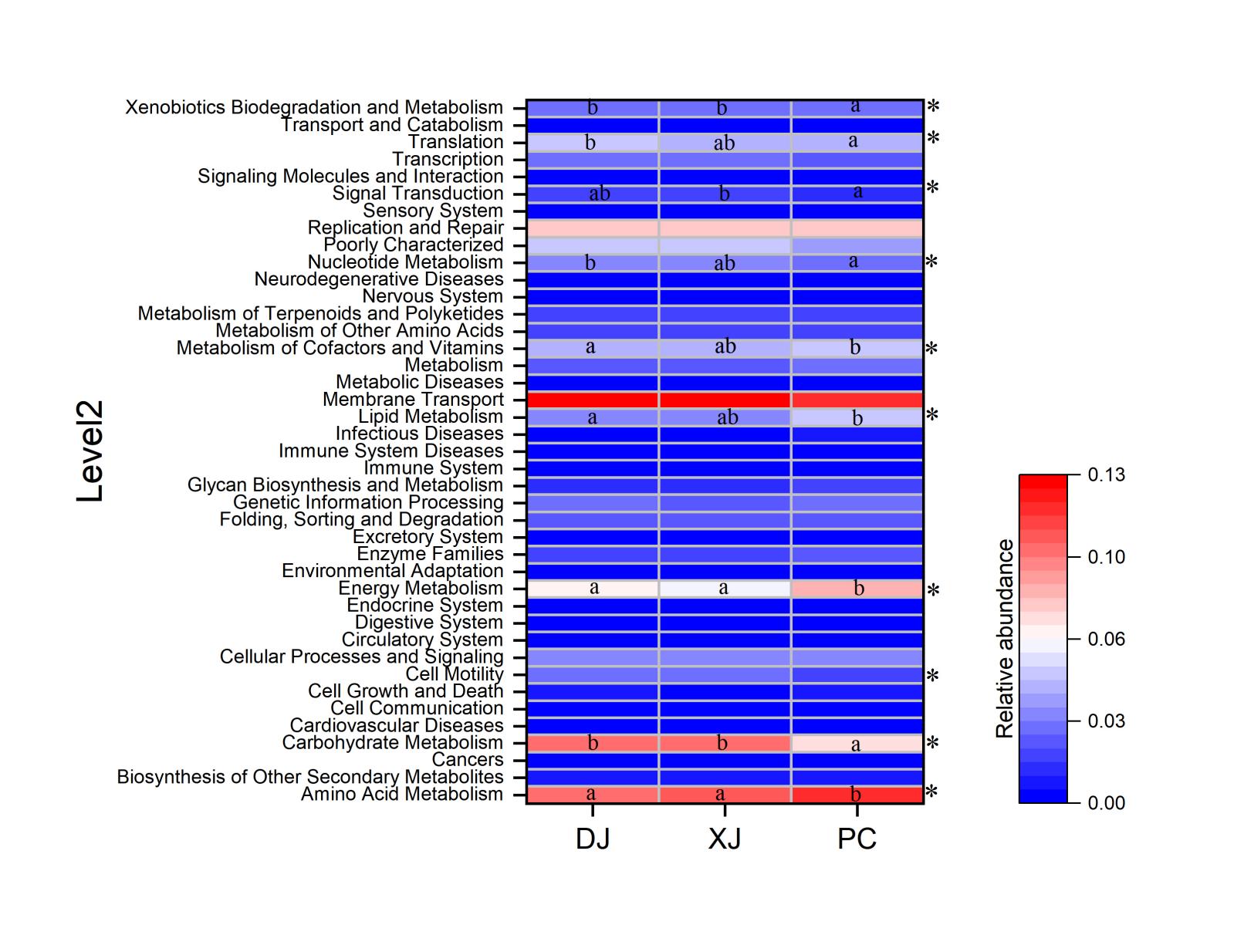
**

Figure S3 KEGG categories derived from the 16S rRNA sequences of the fish gut microbiomes by PICRUSt. Heatmap presenting the relative abundance of gutmicrobial gene functions (level 2) among the three different groups. Samples marked by an asterisk (*) indicate significant differences (*P* <0.05) among the three groups. Samples marked different capital letters indicate significant differences (a < b; *P* < 0.05) among different groups.

Table S1 Selected environmental characteristics for the three groups of redbelly tilapia

| Parameter | Groups | | |
| --- | --- | --- | --- |
|  | DJ | XJ | PC |
| Temperature (℃) | 25.8±1.26 | 25.1±1.06 | 25.3±0.52 |
| pH | 8.4±0.64 | 7.8±0.82 | 6.9±0.35 |
| dissolved oxygen (mg/L) | 7.59±0.89 | 7.85±0.97 | 7.05±0.76 |
| Total nitrogen (mg/L) | 2.13±0.25 | 1.63±0.21 | 2.05±0.16 |
| Total phosphorus (mg/L) | 0.32±0.05 | 0.16±0.04 | 0.42±0.06 |

Table S2 Co-occurrence network results for the gut microbial community of tilapia zillii in the various habitats

|  | Sample |  |  |
| --- | --- | --- | --- |
| Co-occurrence network parameters | DJ | XJ | PC |
| Similarity threshold | 0.6 | 0.6 | 0.6 |
| Nodes | 406 | 376 | 207 |
| Edges | 3650 | 5350 | 1657 |
| Average weighting | 8.988 | 14.229 | 5.903 |
| Network diameter | 8 | 9 | 7 |
| Density | 0.029 | 0.038 | 0.022 |
| Average geodesic distance | 1.908 | 2.832 | 2.907 |
| Modularity | 0.776 | 0.682 | 0.665 |
| Average clustering coefficient | 0.377 | 0.369 | 0.322 |
